# Diversity of pili-specific bacteriophages: genome sequence of IncM plasmid-dependent RNA phage M

**DOI:** 10.1186/1471-2180-12-277

**Published:** 2012-11-24

**Authors:** Janis Rumnieks, Kaspars Tars

**Affiliations:** 1 Biomedical Research and Study Centre, Ratsupites 1, Riga, LV-1067, Latvia

**Keywords:** *Leviviridae*, RNA phage, Pili-specific phage, IncM, Conjugative plasmid, Lysis

## Abstract

**Background:**

Bacteriophages of the *Leviviridae* family are small RNA viruses with linear, positive-sense, single-stranded RNA genomes that encode only four proteins. All phages of this family require bacterial pili to attach to and infect cells. *Leviviridae* phages utilizing F-pili for this purpose have been extensively studied. RNA phages specific for conjugative plasmid-encoded pili other than that of plasmid F have been isolated, but are much less understood and their relation to the F-pili-specific phages in many cases is not known.

**Results:**

Phage M has the smallest known *Leviviridae* genome to date and has the typical genome organization with maturation, coat and replicase genes in the 5′ to 3′ direction. The lysis gene is located in a different position than in other known *Leviviridae* phages and completely overlaps with the replicase gene in a different reading frame. It encodes a 37 residue long polypeptide that contains a transmembrane helix like the other known lysis proteins of leviviruses. Sequence identities of M proteins to those of other phages do not exceed 25% for maturation protein, 51% for coat protein and 41% for replicase. Similarities in protein sequences and RNA secondary structures at the 3′ untranslated region place phage M together with phages specific for IncP, IncC and IncH, but not IncF plasmid-encoded pili. Phylogenetic analysis using the complete genome sequences and replicase proteins suggests that phage M represents a lineage that branched off early in the course of RNA phage specialization on different conjugative plasmids.

**Conclusions:**

The genome sequence of phage M shows that it is clearly related to other conjugative pili-specific leviviruses but has an atypical location of the lysis gene. It provides a better view on the remarkable diversification of the plasmid-specific RNA phages.

## Background

Bacteriophages of the *Leviviridae* family are small viruses that infect several genera of Gram-negative bacteria. They have linear, positive-sense, single-stranded RNA genomes about 3500 – 4200 nucleotides in length that encode only four proteins. All *Leviviridae* phages have three genes in common – maturation, coat and replicase [1]. The replicase cistron encodes the catalytic subunit of the RNA-dependent RNA polymerase complex, which is assembled together with several bacterial proteins [2,3] and replicates phage RNA. The coat protein forms dimers, 90 of which assemble in a *T*=3 icosahedral capsid about 27 nm in diameter and encapsidate the genome [4]. A single copy of the maturation protein binds to phage RNA [5] and gets incorporated into capsids along with it. It is required for infectivity of the virions – the maturation protein binds to bacterial pili, then leaves the capsid and enters the cell as an RNA-protein complex [6].

Many of the *Leviviridae* phages are divided in two genera – leviviruses and alloleviviruses. The major distinction of alloleviviruses is the presence of a minor coat protein A1 in their capsid which is produced by ribosomal read-through of a leaky termination codon of the coat gene [7]. The other difference is that the maturation protein of alloleviviruses also triggers cell lysis [8,9], whereas leviviruses encode a dedicated small lysis polypeptide for this purpose [10-12].

The ssRNA phages that infect *Escherichia coli* cells by adsorbing to F plasmid-coded pili were the first isolates of the *Leviviridae* family [13,14], and to date these “male-specific” phages, with type species MS2 and Qβ, have been the most intensively studied and best characterized of this family. However, the F plasmid is just one of the many conjugative plasmids that are present in nature. These plasmids are often highly divergent from F and are most often grouped according to their mutual compatibility. In *Enterobacteriaceae*, the conjugative plasmids form more than 20 different incompatibility (Inc) groups which are denoted by capital Latin letters [15]. All these plasmids encode conjugative pili, but the pilin subunits often share no similarity.

Several ssRNA phages specific for conjugative pili other than that of plasmid F have been discovered. Phage PRR1 [16] which adsorbs specifically to IncP plasmid-encoded pili was the first such example, and later other phages specific for Inc group C [17], D [18], H [19,20], I [21], M [22] and T [23] plasmids followed. Phages PRR1, C-1 (IncC-specific) and Hgal1 (IncH-specific) have been sequenced [24,25] and phage PRR1 capsids have also been crystallized [26], but no research has been done on the other plasmid-specific phages since their isolation.

The IncM plasmid-specific RNA phage M [22] was isolated from sewage in Pretoria, South Africa in the beginning of the 1980s. IncM plasmids have a broad host range, code for rigid pili and transfer efficiently only when bacteria are growing on solid media [27]. Likewise, the phage is able to propagate in different strains of *Escherichia*, *Salmonella*, *Klebsiella*, *Proteus* and *Serratia*, provided they contain an IncM plasmid. To obtain more insight in plasmid-specific RNA phages, we determined the genome sequence of phage M and present here its analysis and comparison to the genomes of other RNA phages of the *Leviviridae* family.

## Results and discussion

### Overall structure of the genome

**Figure 1:**
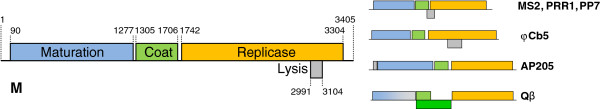
**Genome organization of phage M.** Start and end positions of phage genes are indicated. For comparison, the other known genome organizations of *Leviviridae* phages are represented on the right with genes color-coded as in the M genome. In phage Qβ, protein A1 (bright green) is an extended read-through variant of the coat protein and the lysis function is performed by the maturation protein.

The genome of phage M is 3405 nucleotides long and follows the canonical *Leviviridae* genome organization with maturation, coat and replicase cistrons following each other in the 5′-3′ direction (Figure 1). An unusual feature of the genome is that the lysis gene appears to be located in a different position than in other leviviruses, as discussed below. It is also the smallest known *Leviviridae* genome to date, about 60 nucleotides shorter than that of the group II F-specific phage GA [28]. The protein coding regions of phage M are of similar length to those of phage GA, with maturation and coat genes being a bit longer and replicase somewhat shorter; the greatest savings in M’s genome come from terminal untranslated regions (UTRs), the 5′ UTR being about 45 nucleotides and the 3′ UTR about 20 nucleotides shorter.

### Identification of the lysis gene

All members of the levivirus genus encode a short polypeptide that mediates cell lysis. Amino acid sequences of lysis proteins show great variation and their only unifying feature is the existence of a hydrophobic transmembrane helix within the protein [29]. Lysis proteins have been shown to accumulate in the bacterial membrane where they presumably form pores that lead to cell lysis [30]. In all of the known *Enterobacteria*-infecting leviviruses, the lysis gene overlaps with coat and replicase genes in a different reading frame and is translationally coupled with the coat gene [1]. However, in the genome of phage M, no candidate ORFs at this location could be identified: in the +2 frame relative to the coat gene there are no termination codons until the start of replicase and in the +1 frame only a 17 amino acid long ORF that would encode a non-hydrophobic peptide is found.

**Figure 2:**
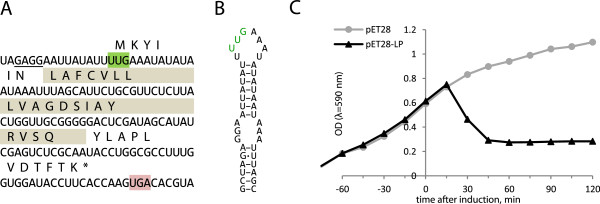
**Lysis protein of phage M.** (**A**) The lysis gene. The Shine-Dalgarno sequence is underlined and initiation and termination codons are indicated by green and pink shading, respectively. The translated amino acid sequence is given above the RNA sequence and the putative transmembrane helix is shaded gray. (**B**) An RNA hairpin around the initiation codon of the lysis gene. The initiation codon and the Shine-Dalgarno sequence are indicated. (**C**) Verification of the lysis gene. Growth of *E.coli* cells harboring either empty vector (pET28) or a plasmid with the cloned lysis gene (pET28-LP) before and after the induction of protein synthesis is shown.

Up to now, there have been two reported cases in the *Leviviridae* family where the lysis gene in is in a different location: *Acinetobacter* phage AP205 has a short lysis gene preceding the maturation gene [31], while *Caulobacter* phage ϕCb5 codes for a longer, two-helix protein that completely overlaps with the replicase gene [32]. To test the possibility that phage M also has a non-canonical localization of the lysis gene, we utilized the fact that the pJET1.2 plasmid, where the cDNA copies of the genome were cloned for sequencing, contains a T7 promoter that can be used to transcribe the insert. Several clones with inserts in the correct orientation with respect to the T7 promoter were selected and transformed to a T7 polymerase-producing *E.coli* strain. When the expression of T7 polymerase was induced, a clone containing an approximately 1000 nucleotide long fragment spanning nucleotides 2098-3129 of the phage genome resulted in a clear cell lysis. Examination of this sequence located a likely candidate for the lysis gene between nucleotides 2991-3104 (Figure 2A). This was based on several criteria: (1) it was the only ORF in the fragment with a significant length (37 amino acids; the shortest known *Leviviridae* lysis protein is that of phage AP205 with 34 amino acids); (2) according to the TMHMM server [33], the ORF-encoded protein was predicted to contain a transmembrane helix with over 95% probability; (3) although the ORF had an unusual initiation codon UUG, there was a rather strong Shine-Dalgarno (SD) sequence GAGG nine nucleotides upstream; (4) RNA secondary structure prediction using the RNAfold server [34] revealed that the initiation codon of the ORF is located on top of an AU-rich stem-loop that would presumably have sufficiently low thermodynamic stability to promote the initiation of translation [35] (Figure 2B). To verify the lytic function of the gene, the ORF together with the original SD sequence and UUG initiation codon was cloned in an inducible protein expression vector. Induction resulted in almost complete cell lysis some 45 minutes after (Figure 2C), thus demonstrating that the approximately 150 nucleotide long stretch is sufficient to encode a functional lysis protein. The abovementioned evidence therefore lets us suggest with some confidence that this is the actual lysis gene of phage M.

### Protein similarities to other phages

The maturation proteins are very variable in *Leviviridae* phages, which is unsurprising given the vast diversity of pili they have evolved to bind. The maturation protein of phage M is most similar to those of the other plasmid-specific RNA phages, but the sequence identity is only 24.5% to phage PRR1, around 22% to C-1, Hgal1, GA and MS2 and drops to 17% when compared to alloleviviruses SP and Qβ. The coat proteins are more conserved and here M groups clearly with phages PRR1, C-1 and Hgal1 with amino acid identities of 48-51%. The identity with F-specific phages is significantly lower and ranges from 27.1% for group II levivirus KU1 to 19% for group IV allolevivirus NL95. Notably, M coat protein shares 24.6% amino acids with that of *Pseudomonas* phage PP7, which is the only plasmid-independent phage for which the sequences could be reasonably aligned. For replicase, the trend is similar as for the maturation protein: the replicase of phage M most resembles that of PRR1 with 41% amino acid identity, followed by other plasmid-dependent phages C-1, Hgal1, MS2 and GA (33-37% identity) and alloleviviruses (27-29% identity). Again, M replicase turns out to be more closely related to that of phage PP7 (25.5% identity) than to the other plasmid-independent phages AP205 and ϕCb5 (17.7 % identity).

### Conserved RNA secondary structures

**Figure 3:**
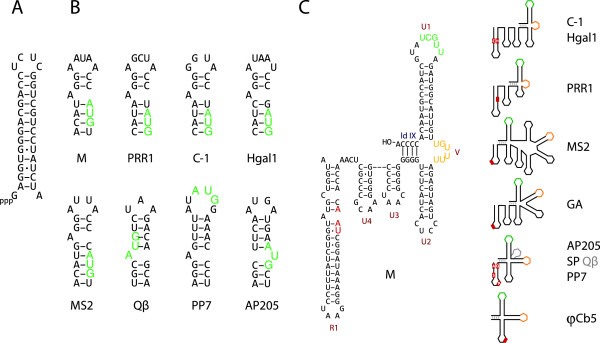
**RNA secondary structures in M genome.** (**A**) A stable hairpin at the very 5′ end of the genome important for phage RNA replication. (**B**) The operator hairpin around the initiation codon of replicase. The analogous hairpins from other *Leviviridae* phages are shown for comparison. Start codons of the replicase gene are colored green. (**C**) Structure of the 3′ untranslated region. The termination codon of replicase is colored dark red, the unpaired stretch corresponding to loop V or V2 in other phages in orange and the conserved nucleotide sequence in the loop of hairpin U1 that potentially forms a long-distance pseudoknot in green. On the right, schematic representations of 3′ UTRs from other phages based either on published data [31,32,45,46] or RNA secondary structure predictions are given for comparison. The 3′ UTR of phage Qβ is closely similar to that of phage SP except for a short extra helix which is depicted in gray. The locations of replicase gene termination codons are represented as red boxes. RNA secondary structures were predicted by the RNAfold server [34].

With the growing number of *Leviviridae* genomes that have been sequenced it has become clear that besides encoding proteins, the secondary and tertiary structure of the RNA itself is also very important. The complex structure of RNA provides binding sites for phage proteins [36-38], regulates their translation [1] and promotes genome packaging in capsids [39]. In many cases where nucleotide stretches from different phage genomes show no sequence similarity, the secondary structures they fold into are nevertheless well preserved. One such example lies at the very 5′ end of all of the sequenced ssRNA phage genomes, where there is a stable GC-rich hairpin that has been suggested to play an important role in phage RNA replication [40]. Phage M is no exception (Figure 3A). Another important RNA structure lies around the initiation codon of replicase. This approximately 20-nucleotide-long stretch folds into a hairpin structure that specifically binds the phage coat protein. This interaction acts as a translational operator to repress synthesis of replicase when enough coat protein accumulates [37] and has been suggested to play also a role in initiating specific encapsidation of the genomic RNA [41]. When the operator hairpin of phage M is compared to those of other ssRNA phages, it is evident that it groups with the conjugative pili-dependent phages PRR1, C-1, Hgal1 and MS2 (Figure 3B). An adenine residue in the loop four nucleotides upstream of the replicase initiation codon and an unpaired purine residue in the stem which are critical for RNA-protein binding in phages MS2 [42], GA [43] and PRR1 [44] are preserved also in phage M, therefore the mechanism of interaction is probably similar.

It is also interesting to take a look at the 3′ untranslated region of the phage genome. The configurations of 3′ UTRs vary between different phages, but nevertheless some similarities exist. In all known *Leviviridae* phages a long-distance interaction designated ld IX bridges the very 3′ terminus with a complementary nucleotide stretch upstream, forming the 3′ terminal domain [45]. The domain usually consists of at least three hairpins, denoted U1, U2 and V. In phage M, the 100-nucleotide-long 3′ UTR is made up from four hairpins U4, U3, U2 and U1 (Figure 3C). In all ssRNA phages the 3′-terminal helix U1 has a remarkably conserved nucleotide sequence in the loop: UGCUU in phages as diverse as MS2, SP and AP205, UGCUG in ϕCb5 and CGCUC in PP7. In the case of Qβ, this loop forms a long-distance pseudoknot with a complementary sequence approximately 1200 nucleotides upstream that is essential for phage replication [47]. In phage M, the sequence of the U1 loop is AUUGCUAUG. It has not been experimentally verified that phages other than Qβ have the pseudoknot, but in M genome a sequence AGCAA is found in the replicase gene some 1215 nucleotides upstream that could potentially basepair with UUGCU in the loop. The other notable feature of the 3′ domains, although less pronounced, is hairpin V (designated V2 in some phages) which in phages MS2, Qβ, SP and AP205 contains a large, adenine-rich loop. There is some evidence that in MS2 this might be one of the sites where the maturation protein binds to the RNA [36]. In phage ϕCb5, however, the candidate hairpin V lacks analogous features and in phages PRR1, C-1 and Hgal1 it does not seem to exist at all; instead, there is a stretch of unpaired nucleotides (UAUAAACA in PRR1, UAUA in Hgal1 and UUAAU in C-1) that connects hairpins U2 and U1 and might serve the same function as hairpin V in other phages. In phage M the situation is similar, but the loop sequence is UUUUGU and contains no adenine residues. When the overall structures of 3′ UTRs from different phages are compared (Figure 3C, right), it is evident that in the distantly related phages ϕCb5, AP205, PP7 and SP the 3′ domain is remarkably simple with just three hairpins, while it is considerably expanded in the plasmid-specific leviviruses, culminating in seven hairpins in phage MS2. In this respect, phages M, C-1, Hgal1 and PRR1 form their own group where the 3′ UTR adopts a characteristic fold of only two hairpins between the ld IX, a stretch of unpaired nucleotides instead of hairpin V and one or two hairpins between the terminal replicase hairpin R1 and ld IX.

### Evolutionary considerations

In many aspects, phage M is a typical representative of the *Leviviridae* family that is clearly related to other conjugative pili-dependent RNA phages. The feature that makes it unique though is the unusual location of its lysis gene. Although there are precedents of this in the distantly related phages AP205 and ϕCb5, it is a bit surprising to find such phenomenon also within a group of otherwise rather closely related phages. Apparently, it is relatively easy for a short ORF encoding a transmembrane helix that causes cell lysis to appear by random mutations, as several phages have arrived at the same mechanism independently. It would also suggest that the location of the lysis gene at this position is probably limited to the IncM plasmid-specific leviviruses or even to a smaller subgroup of these phages. Since M is the only IncM plasmid-specific RNA phage that has been isolated, it is not possible to address this question presently.

**Figure 4:**
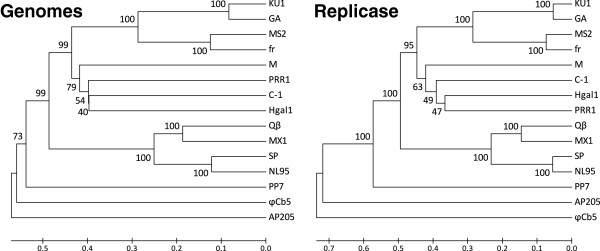
**Phylogeny of RNA phages.** The phylogenetic analysis was based on the complete genomic RNA sequences (left) and amino acid sequences of the replicase (right) which is the most conserved of all ssRNA phage proteins. Trees were constructed by unweighted pair group method with arithmetic mean (UPGMA) and tested using the bootstrap method with 500 replicates. The bootstrap values are expressed as percentages next to the nodes. RNA and protein sequences were aligned using MUSCLE [49] and the phylogenetic trees were constructed in program MEGA5 [50].

The high mutation rates and resulting sequence variability in RNA viruses makes reconstruction of their evolutionary history not a trivial task. Based on similarities between maturation and replicase proteins, phage M seems more related to phage PRR1, while coat protein sequences and structures of the 3′ UTRs suggest that it might be closer to phages C-1 and Hgal1. To further address this question we conducted a phylogenetic analysis of 15 representative *Leviviridae* phages using both the complete genome sequences and also the replicase protein sequences since the RNA-dependent RNA polymerases are the most conserved proteins of all positive-sense RNA viruses [48]. Both trees (Figure 4) confirm that phage M is more closely related to the IncC, IncH and IncP than to the IncF plasmid-dependent phages but they show differences in the clustering of the non-F plasmid specific phages. Although phylogenetic analysis of the coat proteins (not shown) gives the same (M(C-1(Hgal1,PRR1))) clustering as the replicase, low bootstrap values for the IncC, IncH and IncP branches indicate that confidence in that particular branching order is not high and suggest that phages C-1, Hgal1 and PRR1 have radially diverged from a similar ancestral sequence. In both trees phage M represents a lineage that branched off early in the course of specialization on different plasmids after the separation of the IncF lineage had occurred but before the diversification on IncC, IncH and IncP plasmids took place. Both trees also support the idea that the allolevivirus lineage separated from the leviviruses before the specialization on different conjugative pili had occurred and that these phages arrived at the ability to bind to F-pili via an independent evolutionary path.

Although all *Leviviridae* phages use pili for attachment, there is a marked difference between the types of pili they utilize. The type IV pili used by phages AP205, ϕCb5 and PP7 are produced via a genome-encoded type II secretion pathway [51], whereas the plasmid-borne conjugative pili that the other phages utilize belong to a type IV secretion system [52]. Both systems share some functional similarities, like a retractable pilus and a membrane pore, but are thought to have evolved independently [53]. Therefore a jump from one to the other type of pili had to occur at some point in the *Leviviridae* history. Our phylogenetic analysis suggests that the ancestral phage infected cells via type IV pili, like phages AP205, ϕCb5 and PP7 are doing today and a PP7-like virus then might have evolved the ability to bind to some kind of conjugative pili and still sustain infectivity. Consequently, all of the specialized plasmid-dependent RNA phages we know today would be descendants of this ancestral virus.

## Conclusions

We have determined and characterized the genome sequence of IncM plasmid-dependent phage M and shown that it resembles the plasmid-specific leviviruses in many ways but has an atypical location of the lysis gene. It is a valuable addition to the growing number of sequenced *Leviviridae* genomes and provides a better view on the diversity and evolution within this phage family.

## Methods

### Phage propagation and purification

Bacteriophage M and its host *E.coli* J53(RIP69) were obtained from Félix d'Hérelle Reference Center for bacterial viruses, Laval University, Quebec, Canada (catalog numbers HER218 and HER1218, respectively). J53(RIP69) cells were grown in LB medium containing 6 μg/ml tetracycline overnight at 37 °C without agitation. To propagate the phage, 0.5 ml of the host cell suspension and 10 μl of phage lysate (approximately 10^10^ pfu/ml) were spotted on 1.5% LB agar plates, overlaid with 15-20 ml of molten 0.7% LB agar cooled to 45 °C, mixed by swirling and incubated overnight at 30 °C. The next morning, top agar layers from several plates were scraped off, transferred to centrifuge tubes and centrifuged for 20 minutes at 18500 g. Supernatant was collected and phage particles were precipitated by addition of sodium chloride and PEG 6000 to concentrations of 0.5M and 10%, respectively, and incubation for 30 minutes at 4 °C. After centrifugation for 10 minutes at 18500 g, the supernatant was discarded and the pellet was resuspended in a small volume of distilled water. The phage preparation was then layered on top of a preformed five-step cesium chloride gradient (equal volumes of CsCl solutions in 20 mM Tris-HCl pH 7.5 with densities of 1.7, 1.6, 1.5, 1.4 and 1.3 g/ml) and centrifuged for 17 hours in a SW 40Ti rotor at 24000 rpm. 0.5 ml fractions were collected from the top of the gradient and the peak fractions containing phage were pooled and dialyzed against one liter of 20 mM Tris-HCl pH 7.5 overnight at 4 °C. The preparation was concentrated to 500 μl using Amicon Ultra 10K MW cutoff spin unit (Millipore) and used for RNA extraction.

### Isolation of genomic RNA and sequencing

500 μl of purified phage preparation was mixed with 500 μl of phenol and SDS was added to a final concentration of 0.5%. The mixture was vigorously vortexed for 60 s and centrifuged at 12000 g for 3 minutes. The aqueous phase was extracted two more times with a 1:1 phenol/chloroform mixture and once with chloroform. The RNA in the final aqueous phase was precipitated with ethanol, centrifuged and the pellet redissolved in a small volume of DEPC-treated water.

4 μg of the purified RNA was reverse-transcribed with RevertAid Premium reverse transcriptase (Fermentas) using primer 5′-GCAAATTCTGTTTTATCAGACNNNNNN-3′. Reaction products were purified using GeneJet PCR purification kit (Fermentas) and eluted in 20 μl of water. The 3′ termini of the purified first strand cDNAs were dATP-tailed using terminal deoxynucleotidyl transferase (Fermentas). The reaction products were again purified using the PCR purification kit and used as a template for second-strand PCR with primers 5′-GCAAATTCTGTTTTATCAGAC-3′ and 5′-GCGCG(T)_18_-3′ and Pfu DNA polymerase (Fermentas). Reaction products were separated in a 1% agarose gel and a slice corresponding to 1000 – 3000 base pair DNA fragments was cut out. The fragments were extracted using GeneJet gel extraction kit (Fermentas) and ligated in pJET1.2/blunt vector (Fermentas).

Insert-containing clones were sequenced on an ABI Prism 3100 Genetic Analyzer using BigDye Terminator v3.1 kit (Applied Biosystems). Based on the obtained sequence data, additional reverse transcription-PCRs were performed using specific primers to fill gaps and increase coverage. Since the initial cloning procedure already involved 3′-tailing of cDNAs, it was possible to determine the 5′ end of the genome from these clones. To determine the sequence of the 3′ end, phage RNA was tailed with *E.coli* Poly(A) polymerase (Ambion), followed by reverse transcription with primer 5′-GCGCG(T)_18_-3′ and PCR using primers 5′-GCGCG(T)_18_-3′ and 5′-CTGGCGCCTTTGGTGGATAC-3′ corresponding to nucleotides 3072-3091 of the phage genome. Genome assembly and ORF prediction was done with the program ContigExpress from the VectorNTI Suite (Invitrogen).

The genome sequence was deposited in GenBank with accession code JX625144.

### Cloning and expression of the lysis gene

The putative lysis gene was PCR-amplified from a suitable cDNA clone using primers 5′-ATATTCTAGACGAAGGAACAACCATTGCCG-3′ and 5′-TATGAAGCTTACTTGGTGAAGGTATCCACC-3′, the fragment was digested with XbaI and HindIII and ligated into XbaI-HindIII-digested pET28a vector (Novagen), yielding plasmid pET28-LP. To test for the lytic function of the protein, pET28-LP-containing *E.coli* BL21 AI cells (Invitrogen) were grown in LB medium supplemented with 30 μg/ml kanamycin and protein production was induced by adding arabinose to a final concentration of 0.2% and IPTG to a final concentration of 1 mM.

## Competing interests

The authors declare that they have no competing interests.

## Authors’ contributions

JR propagated and purified the phage, sequenced the genome, cloned the lysis gene, analyzed the genome and wrote the paper. KT supervised the work, analyzed the genome sequence and wrote the paper. Both authors read and approved the final manuscript.
